# Efficacy of Bilastine, Dextromethorphan, and Phenylephrine Syrup in Patients With Dry Cough: A Phase 3 Randomised Trial

**DOI:** 10.7759/cureus.75836

**Published:** 2024-12-16

**Authors:** Rahul Kodgule, Pankaj Magar, Sachin K Shivnitwar, Wen Wu, Amol Pendse, Sheldon Creado, Saiprasad Patil, Hanmant Barkate, Monika Tandon

**Affiliations:** 1 Research and Development, Glenmark Pharmaceuticals Limited, Mumbai, IND; 2 Pulmonology, Pulse Multispecialty Hospital, Pune, IND; 3 General Medicine, Lifepoint Multispecialty Hospital, Pune, IND; 4 Global Medical Affairs, Glenmark Pharmaceuticals Limited, Mumbai, IND; 5 Global Medial Affairs, Glenmark Pharmaceuticals Limited, Mumbai, IND

**Keywords:** allergy, bilastine, common cold, cough, dextromethorphan, phenylephrine

## Abstract

Background

Cough in common cold is often associated with rhinorrhoea and nasal congestion, requiring treatment with a cough suppressant, decongestant, and antihistamine. Bilastine is a non-sedating antihistamine, a preferred option over sedating antihistamines. A combination of bilastine, dextromethorphan, and phenylephrine is expected to provide non-sedating treatment for cough associated with a common cold or allergy.

Methods

In this open-label, randomized, active-controlled, multicentre, clinical trial, adult and adolescent subjects with acute dry cough received bilastine 6.6 mg/dextromethorphan 20 mg/phenylephrine 10 mg syrup (BDP) thrice daily or Alex^®^ syrup (chlorpheniramine maleate 4 mg/dextromethorphan 20 mg/phenylephrine 10 mg) for seven days. The objective of the study was to evaluate the efficacy and safety of a fixed-dose combination syrup of bilastine, dextromethorphan, and phenylephrine in cough relief in patients with cough associated with a common cold or allergy.

Results

Of 134 randomized subjects, 67 received BDP and the remaining 67 received Alex® syrup. The least-square mean (LSM) standard error (SE) change in cough severity from baseline on day 4 was 37.86 mm (2.012) in the BDP group (p < 0.001), with a mean difference between the two groups of −4.09 mm (95% CI: −9.68, 1.51). The confidence interval was within the non-inferiority (NI) limits of 30 mm, proving non-inferiority to Alex^®^ syrup (p-value for NI < 0.001). The drowsiness score was significantly lower in the BDP group on day 2 (difference −0.46, p = 0.004), day 4 (difference −0.99, p < 0.001), and day 8 (difference −0.97, p < 0.001). No serious or severe adverse event was reported, and adverse events were comparable between the two groups.

Conclusion

Bilastine/dextromethorphan/phenylephrine combination syrup is efficacious, safe, non-sedating, and non-inferior to Alex^®^ syrup in the treatment of acute dry cough due to a common cold or allergy.

## Introduction

Cough is the most common symptom in individuals seeking medical attention. Effective therapy can reduce the duration and severity of a cough, while a lack of effective therapy can convert an acute cough into a subacute or chronic cough. Antihistamines, decongestants, and cough suppressants are commonly used as over-the-counter medications for relief in acute cough [1].

An acute cough is often the most prominent symptom of the common cold, which itself is the most frequent illness afflicting mankind [2]. Cough in the common cold is often associated with rhinorrhoea, nasal congestion, sore throat, fever, headache, and myalgia [3]. A combination of antihistamine, decongestant, and cough suppressant is an attractive option for relief from acute cough associated with common cold or allergy, as it has a holistic effect on allergy, cough, and nasal congestion.

Alex® syrup, containing chlorpheniramine maleate (CPM) as an antihistamine, dextromethorphan hydrobromide as a cough suppressant, and phenylephrine hydrochloride as a decongestant, is approved in India and is a commonly used combination. However, CPM is a first-generation antihistamine and has the potential for sedation as an adverse effect, which may have an impact on a patient’s quality of life and ability to work or drive. Bilastine is non-sedating, does not enhance the effects of alcohol or CNS sedatives, does not impair driving performance, and has at least similar efficacy as other second-generation H1 antihistamines. Positron emission tomography showed that, compared with other second-generation H1-antihistamines, bilastine has the lowest cerebral histamine H1-receptor occupancy [4].

In a randomized, open-label, phase I study in 36 healthy subjects, the bioavailability of once-daily 20 mg bilastine was compared with a thrice-daily 6.6 mg dose of bilastine over a single day. The geometric least-square mean (LSM) plasma AUC0-t and AUC0-∞ of Bilastine were 838.053 ng*hr/mL and 954.185 ng*hr/mL, respectively. The geometric mean T/R% (90% confidence interval) for AUC0-t and AUC0-∞ were 88.42 (84.15-92.91%) and 98.06 (93.63-102.69%), respectively, demonstrating equivalent exposure over 24 hours. The exposure levels of bilastine with thrice-daily dosing were substantially higher than the concentration levels of bilastine required to have 50% inhibition of H1-histamine (IC50), sufficient for a round-the-clock antihistamine effect with bilastine 6.6 mg thrice daily (data on file).

Hence, a new combination syrup containing bilastine 6.6 mg, dextromethorphan 20 mg, and phenylephrine 10 mg per 10 mL (BDP) thrice daily, thereby replacing CPM with bilastine in the Alex® syrup, was developed for relief of cough associated with a common cold or allergy. In this phase 3 clinical trial, we evaluated the efficacy and safety of BDP in comparison with Alex® syrup.

## Materials and methods

Study design and subjects

This was a multi-centre, randomized, parallel-group, active-controlled, open-label, phase 3 clinical trial. This study was conducted at 10 study sites in India. The first subject was recruited on September 7, 2023, and the last subject completed the last visit on October 4, 2023. Eligible subjects were aged 12-65 years with acute cough of less than seven days’ duration at the time of consent, associated with a common cold or allergy. Subjects were required to have a cough severity of at least 60 mm on the Visual Analogue Scale (VAS) at screening. Exclusion criteria included a history of productive cough with excessive secretions, a history of chronic cough, use of oral or intranasal antihistamines or cough suppressants or decongestants within seven days prior to screening, or use of angiotensin-converting enzyme inhibitors (ACEIs) or angiotensin receptor blockers (ARBs) within 14 days of consent. Subjects with significant cardiovascular disease, uncontrolled hypertension, uncontrolled diabetes mellitus, closed-angle glaucoma, hyperthyroidism, prostatic enlargement, or phaeochromocytoma at screening were also excluded.

This study was registered in the Clinical Trials Registry of India (CTRI) with registration number CTRI/2023/09/057263. This study was conducted in accordance with the ethical principles of good clinical practice, including the Declaration of Helsinki. The study protocol and informed consent form were approved by the Drug Controller General of India and appropriate institutional review boards. All adult patients provided written informed consent, and all adolescent subjects aged 12-18 years provided assent while their parents provided informed consent before screening.

Intervention

Eligible subjects were randomly assigned (1:1) using an Interactive Web Response System (IWRS) to receive either a fixed-dose combination syrup of bilastine 6.6 mg, dextromethorphan 20 mg, and phenylephrine 10 mg per 10 mL (BDP) to be administered 10 mL three times daily or the comparator product Alex® syrup (chlorpheniramine maleate 4 mg, dextromethorphan 20 mg, and phenylephrine 10 mg per 10 mL) to be administered 10 mL three times daily. Subjects were randomized on the day of consent. The treatments were administered for seven days. Concomitant use of any other antihistamine, cough suppressant, or decongestant was not permitted. The use of antibiotics and antiviral agents was permitted. Alex syrup and BDP syrup used in this study were supplied by Glenmark Pharmaceuticals Limited (Mumbai, India).

Procedures

Clinic visits were conducted on days 1 (baseline), 4, and 8. Cough severity, frequency, and impact on sleep disruption in the previous 24 hours were assessed using a 100-mm VAS on days 1, 4, and 8. The subject answered the Patient's Global Impression of Change (PGIC), and the investigator answered the Investigator's Global Impression of Change (IGIC) on days 4 and 8 [5]. PGIC and IGIC were calculated based on a 7-point Likert scale (very much improved to very much worse) and compared to the start of the study treatment [6,7]. Subjects also self-reported the severity of nasal congestion using the Nasal Congestion Score on days 1, 4, and 8 and the drowsiness score on the Profile of Mood State (POMS) questionnaire on days 1, 2 (telephonic), 4, and 8. The POMS questionnaire consists of a checklist with 35 adjectives, which the subjects had to rate on a scale ranging from 0 (not at all) to 6 (extremely). Values or scores in POMS for drowsiness (D), activity (A), nervousness (N), depression (d), and anger (a) self-assessments are computed. In this study, the actual POMS questionnaire will not be used, and only drowsiness similar to that evaluated as per the original POMS will be evaluated with scores ranging from 0 (not at all) to 6 (extremely), as the goal is to assess the sedation or drowsiness effect of the two study treatments [8]. The investigator recorded whether the cough symptoms had resolved and whether the subject was completely cured. Safety was assessed in terms of adverse events and 12-lead electrocardiography.

Outcomes

The primary endpoint was a change from baseline to day 4 in cough severity on a 100 mm VAS. Cough severity by VAS was selected as the primary endpoint, as it has been used as a primary endpoint in clinical trials of cough formulations and is a recommended outcome by the European Respiratory Society [2,9].

Secondary endpoints included the drowsiness score on the POMS questionnaire, the proportion of subjects with resolution of cough symptoms, the proportion of subjects with complete clinical cure, PGIC, IGIC, the change in cough frequency and sleep deprivation on a 100 mm VAS, and the proportion of subjects achieving a score of 0 or 1 in the Nasal Congestion Score.

Statistical analysis

A sample size of 134 subjects was estimated to provide a power of 90% at a one-sided significance level of 2.5% to demonstrate non-inferiority (NI) of BDP in comparison with Alex® syrup, with a non-inferiority margin of 30 mm and assumed standard deviation of 50 mm. NI margin of 30 mm is chosen as the minimum clinically important difference (MCID) in VAS for cough severity, which has been demonstrated to be 30 mm [10].

Randomization was done centrally using computer-generated codes and IWRS. The block size was 2. Subjects randomized to the test or comparator group were allocated sequentially numbered (study level) individual subject packs by IWRS. A random allocation sequence was generated by an independent third party; the investigator enrolled participants. Treatment assignment was done using IWRS, which allocated a kit number to be dispensed. All analyses were performed using IBM SAS® software 9.4 (SAS Institute Inc., Cary, NC). The primary endpoint was analysed in the per-protocol population (all randomized subjects who received at least one dose of study medication, completed the study, and did not have any major protocol deviation), and all the secondary efficacy endpoints were analysed in the modified intent-to-treat (mITT) population (all randomized subjects who received a minimum of one dose of the study medication, who did not have any missing baseline measurement, and a minimum of one post-baseline measurement of efficacy for the primary efficacy variable). The adverse events were analyzed in the safety population (all randomized subjects who received at least one dose of study medication).

The primary endpoint was analyzed using the mixed model repeated measure (MMRM) method. Treatment, visit, baseline value, centre, and treatment-by-visit interaction were used as covariates with an unstructured covariance matrix. Secondary endpoints with continuous variables were analyzed similarly using the MMRM method. Secondary endpoints for proportion were analyzed using the chi-square test.

The study was sponsored by Glenmark Pharmaceuticals Limited. The sponsor had a role in study design, data collection, data analysis, data interpretation, and report writing.

## Results

**Figure 1:**
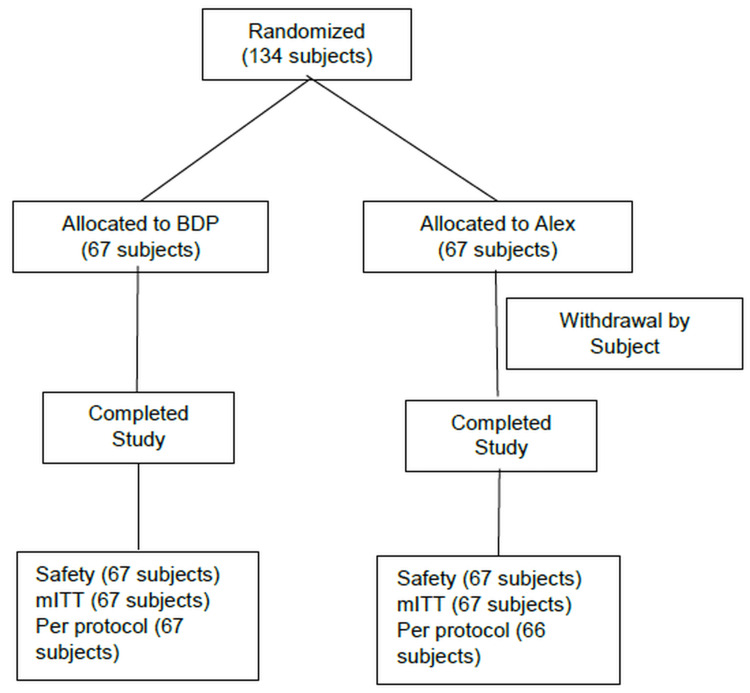
CONSORT diagram BDP: bilastine/dextromethorphan/phenylephrine FDC syrup; mITT: modified intent to treat population.

A total of 134 subjects were randomized to receive study treatment. The safety and mITT population consisted of all 134 subjects, and the per-protocol population consisted of 133 subjects (Figure 1). The demographic and baseline characteristics were comparable between the two treatment groups (Table 1). A total of 15 (22.38%) subjects in the BDP group and 12 (17.91%) subjects in the Alex group were adolescents.

**Table 1:** Baseline characteristics (safety population) BDP: bilastine/dextromethorphan/phenylephrine fixed combination syrup.

Characteristic	Alex® (N=67)	BDP (N=67)
Age, years, mean ± SD	32.1 ± 14.62	33.6 ± 13.78
Age, 12–18 years		
n (%)	15 (22.38)	12 (17.91)
Mean ± SD	15.1 ± 1.87	15.3 ± 1.37
Gender, n (%)		
Male	42 (62.7)	49 (73.1)
Female	25 (37.3)	18 (26.9)
Body weight, kg, mean ± SD	58.7 ± 13.46	59.7 ± 10.95
Height, cm, mean ± SD	159.9 ± 11.23	161.6 ± 8.27
Cough severity on VAS, mm		
Mean ± SD	76.0 ± 7.60	78.1 ± 8.41
Duration of cough, days		
Mean ± SD	3.6 ± 1.14	3.6 ± 1.22

Primary efficacy analysis

**Figure 2:**
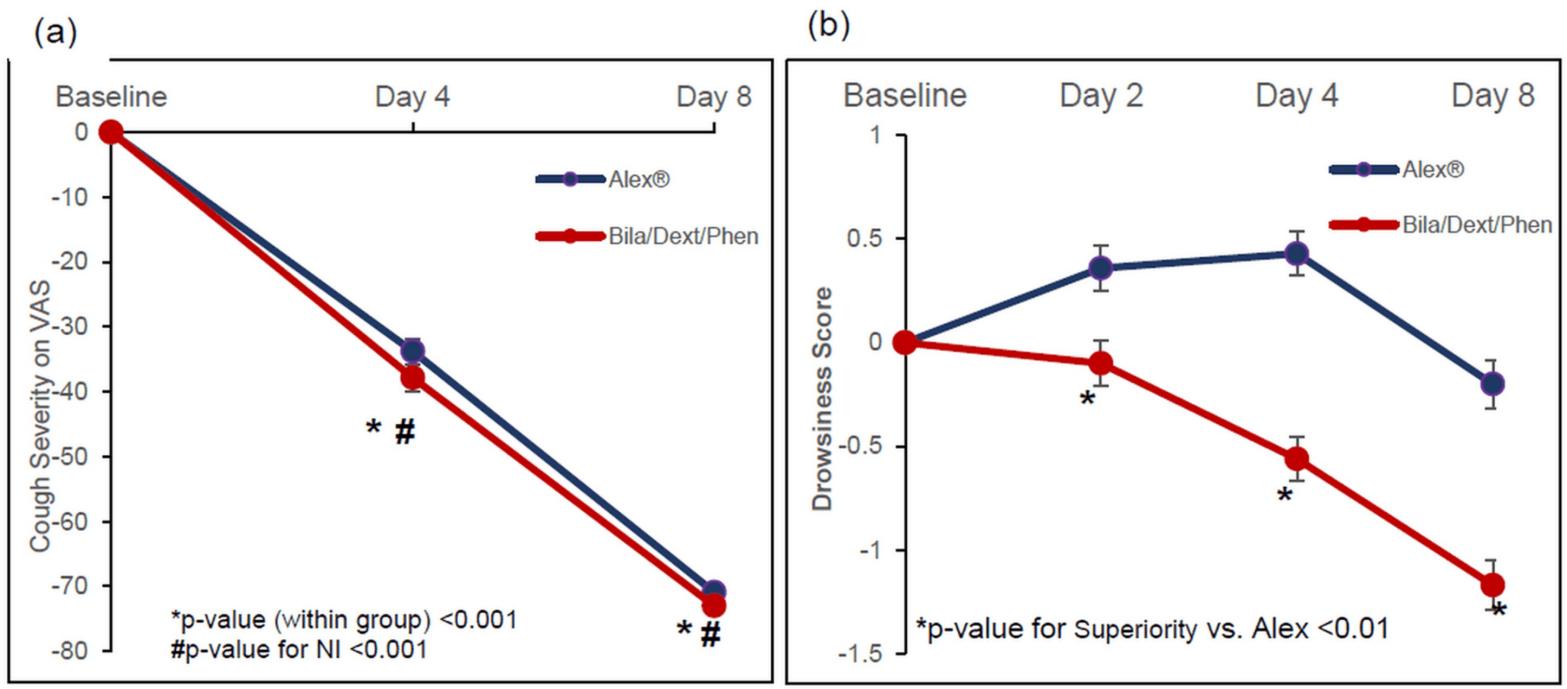
(a) Change from baseline in cough severity (per protocol); (b) change from baseline in Drowsiness Score on Profile of Mood State (POMS) questionnaire (in modified intent to treat population)

The primary efficacy endpoint of change from baseline in cough severity on a 100 mm VAS was analyzed using the MMRM method in the per-protocol population. The LSM (SE) change from baseline in cough severity on day 4 was −37.86 mm (2.012) in the BDP group (p < 0.001). The LSM difference between the two groups was −4.09 mm (95% CI difference: [−9.68, 1.51]). The upper bound of the 95% CI (1.51 mm) was less than the non-inferiority (NI) margin of 30 mm, proving non-inferiority (p-value for NI < 0.001) (Table 2, Figure 2). The same significant results were also obtained in the mITT population. Thus, the primary endpoint was achieved. The same significant results were also obtained on day 8 and in the mITT population.

**Table 2:** Efficacy endpoints, primary and key secondary BDP: bilastine/dextromethorphan/phenylephrine fixed combination syrup; VAS: Visual Analogue Scale; LSM: least square mean; SE: standard error. ^#^Primary endpoint analysed in per protocol population and secondary endpoint analysed in the modified intent to treat population. ^£^Analyzed using the mixed model repeated measure (MMRM) method.

Endpoint	Alex	BDP
Cough severity on a 100 mm VAS, change from baseline^#£^		
Day 4		
LSM	−33.78	−37.86
SE	1.994	2.012
Difference, mean (95% CI)	NA	−4.09 (−9.68, 1.51)
p-value for non-inferiority	NA	<0.001
Day 8		
LSM	−70.88	−73.00
SE	0.787	0.800
Difference, mean (95% CI)	NA	−2.12 (−4.31, 0.07)
p-value for non-inferiority	NA	<0.001
Drowsiness score on profile of mood state, change from baseline^#£^		
Day 2		
LSM	0.36	−0.10
SE	0.110	0.111
Difference, mean (95% CI)	NA	−0.46 (−0.76, −0.15)
p-value of difference		0.004
Day 4		
LSM	0.43	−0.56
SE	0.107	0.107
Difference, mean (95% CI)	NA	−0.99
p-value of difference	NA	<0.001
Day 8		
LSM	−0.20	−1.17
SE	0.116	0.118
Difference, mean (95% CI)	NA	−0.97 (−1.30, −0.64)
p-value of difference		<0.001

At day 8, LSM (SE) change from baseline in cough severity was −70.88 mm (0.787) in the BDP group (p < 0.001). The LSM difference between the two groups was −2.12 mm (95% CI difference: [−4.31, 0.07]), which also met the non-inferiority.

Secondary efficacy analysis

Drowsiness Score on POMS Questionnaire

The Drowsiness Score measured using the POMS Questionnaire was significantly lower in the BDP group compared to the Alex group on days 2, 4, and 8. The LSM difference (SE) between treatments in change from baseline in drowsiness score on days 2, 4, and 8 was −0.46 (0.155), −0.99 (0.150), and −0.97 (0.164), respectively, favouring the BDP group. The p-value for superiority was statistically significant (p = 0.004 on day 2 and p < 0.001 on day 4 and day 8) (Table 2, Figure 2).

Proportion of Subjects With Resolution of Cough Symptoms

The proportion of subjects with resolution of cough symptoms was comparable between the two treatment groups. On day 4, 5 (8.6%) subjects in the BDP group and 3 (5.8%) subjects in the Alex group, and on day 8, 55 (94.8%) subjects in the BDP group and 50 (96.2%) subjects in the Alex group had cough symptoms resolved (Table 3).

**Table 3:** Efficacy endpoints, other secondary (in modified intent to treat population) BDP: bilastine/dextromethorphan/phenylephrine fixed combination syrup; VAS: Visual Analogue Scale; LSM: least square mean; SE: standard error. *Subjects whose cough symptom resolved on day 1 were not included in the analysis. ^$^Subjects who achieved clinical cure on day 1 were not included in the analysis. ^#^Analysis in subjects who had a nasal congestion score of ≥2 at baseline. ^£^Analyzed using the Mixed Model Repeated Measure (MMRM) method. ^€^Analyzed using the chi-square test or Fisher’s exact test.

Endpoint	Alex	BDP
Proportion of subjects with resolution of cough symptoms^€^		
N*	52	58
Day 4		
n (%)	3 (5.8)	5 (8.6)
Day 8		
n (%)	50 (96.2)	55 (94.8)
Proportion of subjects with complete clinical cure^€^		
N^$^	51	58
Day 4		
n (%)	1 (2.0)	5 (8.6)
Day 8		
n (%)	49 (96.1)	55 (94.8)
Patient global impression of change (PGIC)^£^		
N	67	67
Day 4		
LSM (SE)	4.56 (0.074)	4.63 (0.075)
Difference, mean (95% CI)	NA	0.06 (−0.14, 0.27)
Day 8		
LSM (SE)	5.68 (0.057)	5.64 (0.058)
Difference, mean (95% CI)	NA	−0.05 (−0.21, 0.11)
Investigator global impression of change (IGIC)^£^		
N	67	67
Day 4		
LSM (SE)	4.61 (0.062)	4.66 (0.062)
Difference, mean (95% CI)	NA	0.05 (−0.12, 0.22)
Day 8		
LSM (SE)	5.58 (0.059)	5.64 (0.060)
Difference, mean (95% CI)	NA	0.06 (−0.11, 0.22)
Cough frequency on a 100 mm VAS, change from baseline^£^		
N	67	67
Day 4		
LSM (SE)	−32.59 (2.006)	−35.01 (2.007)
Difference, mean (95% CI)	NA	−2.42 (−8.03, 3.19)
Day 8		
LSM (SE)	−65.62 (0.605)	−67.78 (0.616)
Difference, Mean (95% CI)	NA	−2.15 (−3.83, −0.47)
Sleep deprivation on a 100 mm VAS, change from baseline^£^		
N	67	67
Day 4		
LSM (SE)	−30.28 (2.350)	−33.44 (2.353)
Difference, mean (95% CI)	NA	−3.16 (−9.74, 3.41)
Day 8		
LSM (SE)	−56.01 (0.766)	−58.91 (0.782)
Difference, mean (95% CI)	NA	−2.90 (−5.03, −0.76)
Proportion of Subjects achieving a score of 0 or 1 in the Nasal Congestion Score^€^		
N^#^	11	17
Day 4		
n (%)	11 (100.0)	15 (88.2)
Day 8		
n (%)	11 (100.0)	17 (100.0)

Proportion of Subjects With Complete Clinical Cure

A complete clinical cure was defined as the resolution of all symptoms related to cough and associated common cold or allergy. The proportion of subjects who achieved complete clinical cures on days 4 and 8 was comparable between the two treatment groups. On day 4, 5 (8.6%) subjects in the BDP group and 1 (2.0%) subject in the Alex group, and on day 8, 55 (94.8%) subjects in the BDP group and 49 (96.1%) subjects in the Alex group had complete clinical cure (Table 3).

PGIC

There was a statistically significant improvement on day 4 (p < 0.001) and day 8 (p < 0.001) in the PGIC in the BDP group. The difference between the two treatments was not significant (Table 3).

IGIC

There was a statistically significant improvement on day 4 (p < 0.001) and day 8 (p < 0.001) in the IGIC in the BDP group. The difference between the two treatments was not significant (Table 3).

Change From Baseline in Cough Frequency on a 100 mm VAS

There was a statistically significant improvement on day 4 (p < 0.001) and day 8 (p < 0.001) in cough frequency in the BDP group. The LSM (SE) change from baseline in cough frequency on day 4 was −35.01 (2.007) and on day 8 was −67.78 (0.616) in the BDP group (p < 0.001). The difference between the two treatments was small (Table 3).

Change From Baseline in Sleep Deprivation on a 100 mm VAS

There was a statistically significant improvement on day 4 (p < 0.001) and day 8 (p < 0.001) in sleep deprivation in the BDP group. The LSM (SE) change from baseline in sleep deprivation on day 4 was −30.28 (2.350), and on day 8 it was −56.01 (0.766) in the BDP group (p < 0.001). The difference between the two treatments was small (Table 3).

Proportion of Subjects Achieving Score of 0 or 1 in the Nasal Congestion Score

The proportion of subjects achieving a score of 0 or 1 in the Nasal Congestion Score at days 4 and 8 was analyzed in subjects who had a score of ≥2 at baseline. However, only 28 subjects (17 subjects in the BDP group and 11 subjects in the Alex group) had a baseline nasal congestion score of ≥2. The proportion of subjects achieving a nasal congestion score of 0 or 1 on day 4 was 15 (88.2%) in the BDP group and 11 (100%) in the Alex group, while that on day 8 was 17 (100.0%) in the BDP group. The difference between the two treatments was small (Table 3).

Safety and adverse event profile

The study did not report any serious adverse events or deaths. A total of 12 out of 134 randomized subjects (8.95%) in the safety population reported treatment-emergent adverse events (TEAEs). All the AEs were mild to moderate; no severe AE was reported. No AE was considered related to the study drug. No adverse event resulted in study drug termination or subject withdrawal.

Three (4.5%) subjects in the BDP group had TEAEs, and events in all three subjects (4.5%) were mild in intensity. The BDP group did not report any adverse events of moderate or severe intensity. Nine (13.4%) subjects in the Alex group had TEAEs. Eight subjects (11.9%) experienced mild TEAEs, while one subject (1.5%) experienced moderate-intensity events.

The adverse events reported in >1.0% of subjects were abdominal pain, pain, and headache reported by one subject (1.5%) each in the BDP group. The adverse events reported in >1.0% of subjects in the Alex group were headache (5 [7.5%]), asthenia (3 [4.5%]), and abdominal pain and pyrexia reported by one subject (1.5%) each (Table 4).

**Table 4:** Treatment emergent adverse events (safety population)

Adverse event	Alex® (N=67) n (%)	Bila/Dext/Phen (N=67) n(%)
Adverse events	9 (13.4)	3 (4.5)
Serious adverse events	0	0
Death	0	0
Abdominal pain	0	1 (1.5)
Abdominal pain upper	1 (1.5)	0
Asthenia	3 (4.5)	0
Pain	0	1 (1.5)
Pyrexia	1 (1.5)	0
Headache	5 (7.5)	1 (1.5)

## Discussion

This is the first study evaluating a non-sedative cough suppressant combination syrup. This study demonstrated the efficacy and safety of a fixed-dose combination of bilastine 6.6 mg, dextromethorphan 20 mg, and phenylephrine 10 mg per 10 mL (BDP), administered thrice daily in patients with dry cough associated with a common cold or allergy. There was a change from baseline in the Visual Analogue Scale for cough severity of −37.70 on day 4 and −72.97 on day 8 in the BDP group. This reduction is more than the minimum clinically important difference of 30 mm, suggesting a statistically significant and clinically relevant reduction in cough severity [10]. This change was non-inferior to the Alex group on both days 4 and 8. Thus, the primary endpoint was achieved.

The non-sedative feature of BDP compared to Alex® was demonstrated in the POMS questionnaire for sedation. The drowsiness score was significantly lower with BDP compared to Alex®. On days 2, 4, and 8, the drowsiness score on POMS was significantly lower in the BDP group compared to the Alex group, with values of approximately 0.46, 0.99, and 0.97.

The other secondary endpoints - cough frequency, sleep deprivation, nasal congestion score, patient’s global impression of change, and investigator’s global impression of change - were comparable between the BDP and Alex groups.

The study did not report any deaths or serious adverse events. No severe adverse event was reported, and all the adverse events were mild or moderate. Overall, BDP was safe and well tolerated and had an adverse event profile comparable to that of Alex®.

The study demonstrated that BDP has comparable efficacy and safety to the marketed product, Alex®, and has an advantage over Alex® of causing less or no sedation. Bilastine/dextromethorphan/phenylephrine combination syrup may be suitable for patients who require treatment with a non-sedative combination, e.g., school-going children, working professionals, workers handling machinery, automobile drivers, etc.

The results should be interpreted in light of the strengths and limitations of the study. This was a randomized, controlled trial with a sound study design. Alex® was used as a comparator as Alex® is an approved product and has a similar composition as BDP, and Alex® has a large post-marketing experience of many years. The primary endpoint, change from baseline in cough severity measured with a Visual Analogue Scale, is a validated endpoint for cough and has been used in multiple studies in the past. The major limitation was the open-label study design, especially considering the use of a less objectively measured endpoint. The comparisons between BDP and Alex® are consistent across all endpoints and time points, and the differences between the two groups are minimal, with the exception of BDP's lower drowsiness score. This suggests that the use of an open-label design is unlikely to have caused significant bias.

## Conclusions

Bilastine/dextromethorphan/phenylephrine combination syrup is efficacious and safe in the treatment of acute dry cough, common cold, or allergy. The efficacy of bilastine/dextromethorphan/phenylephrine is non-inferior to Alex® syrup in the reduction of cough severity. Based on the results, bilastine/dextromethorphan/phenylephrine is non-sedating compared to Alex®.
